# Epigenetics, Microbiota, and Breast Cancer: A Systematic Review

**DOI:** 10.3390/life14060705

**Published:** 2024-05-30

**Authors:** Alba Soldado-Gordillo, Ana Isabel Álvarez-Mercado

**Affiliations:** 1 Department of Biochemistry and Molecular Biology 2, School of Pharmacy, Campus de Cartuja s/n, 18071 Granada, Spain; albasg15@correo.ugr.es; 2 Department Pharmacology, School of Pharmacy, Campus de Cartuja s/n, 18071 Granada, Spain; 3 Institute of Nutrition and Food Technology “José Mataix”, Biomedical Research Center, Parque Tecnológico Ciencias de la Salud, Avda. del Conocimiento s/n, Armilla, 18016 Granada, Spain; 4 Instituto de Investigación Biosanitaria ibs.GRANADA, Complejo Hospitalario Universitario de Granada, 18071 Granada, Spain

**Keywords:** breast cancer, epigenetic, microbiota, nutrition, stress

## Abstract

Breast cancer is the most frequently diagnosed cancer in women worldwide. According to recent studies, alterations in the microbiota and epigenetic modulations are risk factors for this disease. This systematic review aims to determine the possible associations between the intestinal and mammary microbial populations, epigenetic modifications, and breast cancer. To achieve this objective, we conducted a literature search in the PubMed, Web of Science, and Science Direct databases following the PRISMA guidelines. Although no results are yet available in humans, studies in mice suggest a protective effect of maternal dietary interventions with bioactive compounds on the development of breast tumors in offspring. These dietary interventions also modified the gut microbiota, increasing the relative abundance of short-chain fatty acid-producing taxa and preventing mammary carcinogenesis. In addition, short-chain fatty acids produced by the microbiota act as epigenetic modulators. Furthermore, some authors indicate that stress alters the gut microbiota, promoting breast tumor growth through epigenetic and gene expression changes in the breast tumor microenvironment. Taken together, these findings show the ability of epigenetic modifications and alterations of the microbiota associated with environmental factors to modulate the development, aggressiveness, and progression of breast cancer.

## 1. Introduction

The term cancer refers to a group of diseases in almost any organ or tissue of the body due to the uncontrolled growth of abnormal cells. Histologically, breast cancer is a type of cancer that develops from breast tissue, including adipose tissue, fibrous tissue, and glandular tissue [1].

Breast cancer is the most diagnosed malignancy in women worldwide, generating 2.3 million new cases each year. It is also the second-leading cause of death in this group, with 666,103 deaths registered in 2022 according to the latest epidemiological surveys provided by the Global Cancer Observatory (GLOBOCAN) [2].

**Figure 1:**
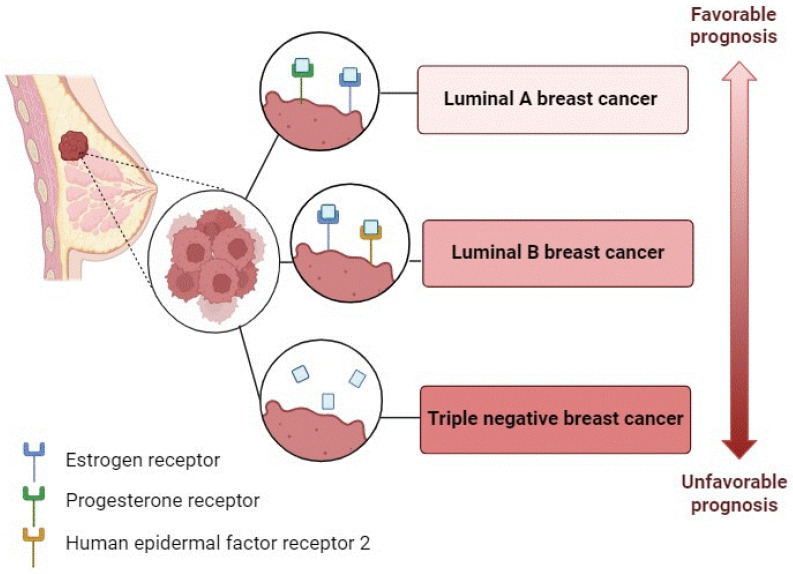
Molecular characterization of breast cancer.

Molecular characterization is crucial in the diagnosis and prognosis of this disease. This type of cancer is molecularly classified according to three main biomarkers: the progesterone receptor, estrogen receptor (ER), and epidermal growth factor receptor 2. The luminal A and luminal B subtypes are ER-positive and constitute about 75% of breast tumors. The triple-negative breast cancer (TNBC) subtype accounts for about 15–20% of breast tumors and lacks all three of the receptors mentioned above [3] (Figure 1). The prognosis for the different cancer subtypes varies from excellent for luminal subtype A to least favorable for TNBC, which has limited treatment options [4].

Nowadays, the treatment of breast cancer is based on four main strategies: surgery, radiotherapy, systemic treatment, and immunotherapy. Radiotherapy can be used as an adjuvant or palliative therapy. Systemic treatment is administered as adjuvant or neoadjuvant and includes chemotherapy, endocrine hormone therapy, and biological or targeted therapy [5].

Breast cancer is a multifactorial disease [6]. Some of the risk factors are passive (those that patients merely experience passively), such as genetic predisposition, and others are active risks that are therefore preventable and modifiable (e.g., dietary patterns, obesity, or stress) [1].

Numerous studies have related several dietary compounds with protective effects against multiple cancers, including breast cancer. For instance, the maternal diet contributes to these benefits in a transgenerational manner [7]. It is believed that events occurring during early development, including maternal nutrition, have an important impact on the health of offspring and the progression of breast cancer [8]. Stress is also an environmental factor that influences the development of breast cancer and aggravates the disease. Chronic stress is detrimental to long-term health because of the constant release of hormones such as cortisol. Given that stress is increasingly inevitable, this factor has become the subject of many studies [9].

In addition to these well-defined risk factors, recent studies suggest that epigenetic modifications and changes in the microbiota may be involved in the development of breast tumors [10].

The microbiota is the set of microbes that reside in our organism. Different microbiota ecosystems are located in various body parts, with the gut microbiota standing out quantitatively [6]. The function of the gut microbiota is to maintain an active balance with the host, performing local and remote tasks in several physiological processes. However, when the balance of this commensal community is disrupted, a phenomenon known as dysbiosis can be involved in the development of various human diseases, including cancer [11].

Everyone’s gut microbiota is unique and is determined by genetic and lifestyle factors (among other factors). This high variation between individuals makes the definition of dysbiosis challenging. Microbial dysbiosis occurs when the microbial community of an organ or tissue is abnormally composed or maladapted and has recently been implicated as a key factor in the onset and progression of cancer. Indeed, some authors have suggested that altering the composition of the gut microbiota may promote the development and aggressiveness of extraintestinal tumors and contribute to the generation of hyperplastic and neoplastic lesions in the mammary glands [12].

Although the gut microbiota has received the most research interest concerning its connection to cancer, other anatomical sites have also been examined, including the mammary glands. Even though initially conceived as a sterile site, it has recently been suggested that the microbial populations of breast tissue may be involved in the initiation and progression of breast cancer [13].

In addition, the microbiota may play a destructive or protective role in the development of breast cancer mediated by epigenetic regulation [8]. Epigenetics consists of various biological processes that affect gene expression, resulting in heritable phenotype or gene activity changes without altering the underlying DNA sequence. Covalent post-translational modifications of histones, DNA methylation, and modification of non-coding RNAs, such as miRNAs, are essential epigenetic mechanisms in biological processes such as cell replication, survival, division, and regulation of gene expression. However, disrupting these epigenetic modulations can lead to the activation of oncogenic transcriptional pathways and alterations in the function of genes implicated in mammary tumor development [14].

While miRNAs can regulate gene expression by degrading multiple mRNAs and interfering with the translation that regulates tumor cell survival and multiplication [15], we will focus on DNA methylation and post-translational modification of histones because of their importance during early mammalian development. DNA methylation consists of adding a methyl group to the fifth carbon position of cytosine, mainly at the cytosine-guanine dinucleotides, through the action of DNA methyltransferase (DNMT) enzymes [16]. On the other hand, histones are proteins susceptible to post-translational modifications, including methylation and demethylation. However, it is their acetylation and deacetylation that have attracted the most interest in the microbiological area of studying breast cancer [16,17].

The individualized epigenome is initiated during early development by establishing unique epigenetic marks through epigenetics reprogramming. These epigenetic signatures persist throughout life and can even be passed on to offspring through germline epigenetic inheritance. This provides a reliable mechanism for transcriptional regulation of genes across generations [8].

It is becoming increasingly clear that the origins of breast cancer can be traced back to early maternal and fetal lifestyles. In contrast to the genome, epigenomes are particularly sensitive to environmental factors and can be dysregulated during early development. One of the environmental stimuli that has the greatest impact on the fetal epigenome is the nutritional status of the mother, in part because maternal nutrition is the only source of nutrients during this period [8].

Intestinal microorganisms can ferment dietary fiber to produce low molecular weight bioactive compounds, such as short-chain fatty acids (SCFAs), which may be involved in epigenetic processes, including at extraintestinal sites. Many advances suggest that dysregulation of the epigenome may also be involved in the pathogenesis of mammary neoplasia [15]. Disruption of the metabolomic profile of gut and blood metabolites has been implicated in this effect. These blood metabolites may subsequently mediate epigenetic and gene expression changes in the breast tumor microenvironment which promote breast cancer development [3].

In this regard, in recent years, the relationship between the gut microbiota and epigenetic DNA modifications and breast cancer has become of great interest in biomedical research [10]. This review aims to identify possible associations between gut and breast microbial populations, epigenetic modifications, and breast cancer risk and progression.

## 2. Materials and Methods

### 2.1. Search Strategy and Selection Criteria

This systematic review is based on the relevant literature in PubMed, Web of Science, and Science Direct databases. These systematic searches were conducted from February 2023 to June 2023, following the Preferred Reporting Items for Systematic Reviews and Meta-Analysis (PRISMA) guidelines. To use a controlled vocabulary, and to make sure that the terminology used is the commonly accepted English terminology to denote the concepts under study, the search strategy was performed using the following Medical Subject Headings (MeSH) terms: (“breast cancer” OR “breast neoplasms” [Title/Abstract] OR “breast tumors” [Title/Abstract]) AND (“microbiota” [Title/Abstract] OR “dysbiosis” [Title/Abstract]) AND (“epigenetics” [Title/Abstract] OR “epigenetic mechanisms” [Title/Abstract] OR “histone post-translational modification” [Title/Abstract] OR “DNA methylation” [Title/Abstract]) AND (“nutrition” [Title/Abstract] OR “maternal nutrition” [Title/Abstract]) AND (“stress” [Title/Abstract]). We included novel articles addressing the association between breast cancer, microbiota, or epigenetic mechanisms. The exclusion criteria included studies not published in English or before January 2019, studies focused on cancers other than breast cancer, and reviews or other works that did not provide original data.

### 2.2. Data Extraction

To extract data from the included studies, we relied on key information that allowed us to find associations between breast cancer, epigenetic mechanisms, and microbial communities in the gut or breast. As mentioned above, we focused on data from breast cancer cases, although some articles also included data from other cancer types. There were no restrictions on the study design. Both in vitro and in vivo tests in animal models and human clinical trials were included. Clinical trial data included both diseased and healthy women of all ages, due to the greater impact of breast cancer on women. Ethnicity and the type of cancer were also not mutually exclusive.

On the other hand, although we are aware of the recent changes in the taxonomy and nomenclature of bacteria, we have retained the nomenclature used by the authors to improve the traceability of the works included in this review.

### 2.3. Quality Assessment

The quality assessment and selection were performed by two authors (A.I.Á.-M. and A.S.G., who independently worked according to the main criteria of Population, Intervention, Comparison, and Outcome (PICO)) (Table 1).

## 3. Results

**Figure 2:**
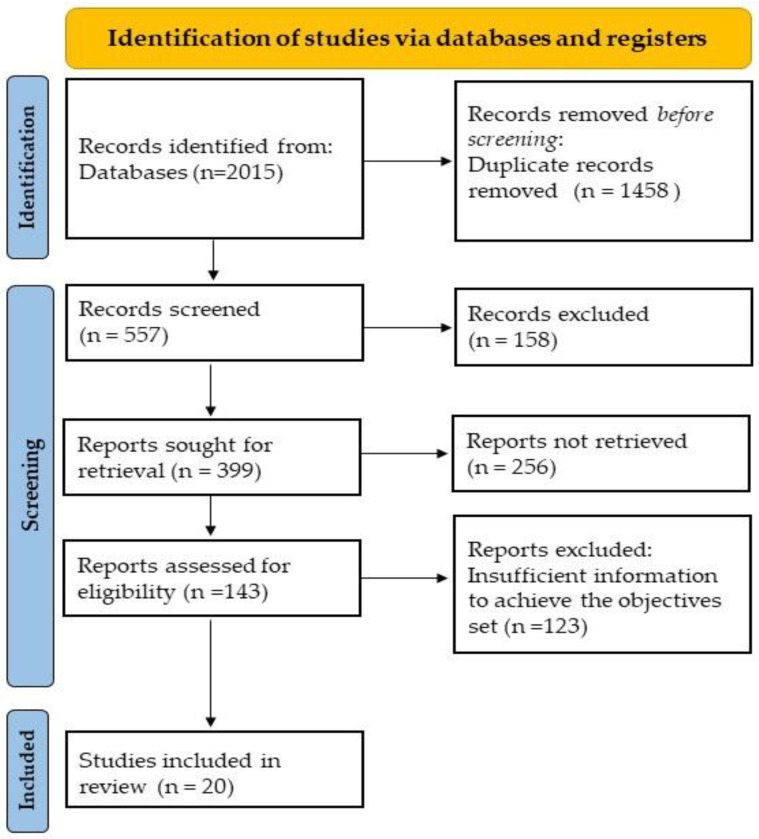
PRISMA flowchart.

A total of 2015 publications were initially identified. After eliminating duplicates and applying the eligibility criteria described above, the search was reduced to 557. To continue the selection, the titles and abstracts of the articles were reviewed to decide whether the information contained in the articles was relevant to the aim of this review. At this stage, 158 articles were excluded. When the relevance of the articles was not clear from the abstract, these studies were selected, and the full text was assessed. In the end, 20 studies were included. The PRISMA flowchart is shown in Figure 2.

### Main Outcomes

The study of parental nutritional status and diet (Table 2) has gained importance in recent years because of its impact on the health of offspring, revealing the relationship between these variables and the occurrence of chronic human disorders and diseases. In line with these findings, some authors pointed to epigenetic mechanisms as a way in which parental nutrition influences offspring disease development in adulthood [18,19].

The results of several studies showed that changes in DNA methylation patterns in breast tissue are diet-dependent, with DNA hypermethylation predominating in the breast tissue of rodents with obesity compared with rodents subjected to calorie restriction, according to a study by Bowers et al. (2022) [20]. Furthermore, research by Li et al. (2020) [21] and Arora et al. (2022) [19] showed the protective effect of maternal feeding enriched in bioactive components such as sulforaphane (SFN) in broccoli sprouts (BSps) against the development of breast cancer in the offspring through significant transcriptional reduction of important enzymes, such as DNMTs and histone deacetylases (HDACs), which are involved in epigenetic modifications. The same protective effect against breast cancer through histone acetylation and DNA methylation was observed in the study by Abbas et al. (2021) [7], which was based on a diet rich in canola oil. Likewise, Chen et al. (2022) [18] showed that the epigenetic protective effect against breast cancer from genistein (GE) in transgenic soy depends on the time of maternal exposure to the dietary component. 

**Table 2:** Summary of the studies addressing nutritional status and epigenetic modifications in breast cancer.

Authors	Study Design	Model	Sample Size	Intervention	Key Findings
Li et al., 2020 [21]	In vivo	Her 2/neu female mice that develop ER (−) tumors	N = 42 broods	Mothers treated with BSps from 3 weeks of age until the weaning of their offspring.	↓ The development of mammary tumors in offspring. Methylated histone H3K9 was enriched in the promoter regions of tumor suppressor genes.
Arora et al., 2022 [19]	In vivo	SV40 female mice that develop ER (−) tumors	N = 40 broods	Mothers treated with BSps from 4 weeks of age until the weaning of their offspring.	Preventive effects of breast cancer in offspring. Histone acetylation and global DNA methylation were affected. SFN downregulated HDAC expression, leading to an increase in histone acetylation.
Abbas et al., 2021 [7]	In vivo	BALB/c female mice	N = 200 broods	Mothers treated with canola oil during gestation and lactation of their offspring.	Epigenetic modifications that contributed to the activation of pathways that suppressed cell proliferation in tumorigenesis, with ↑ survival, ↓ tumor size, and ↓ mortality.
Chen et al., 2022 [18]	In vivo	SV40 female mice that develop mammary tumors spontaneously	N = 30 broods	Mothers treated with GE from 4 weeks of age until the weaning of their offspring.	Tumor demethylation in the progeny.
Chen et al., 2022 [8]	In vivo	Her 2/neu and SV40 female mice that develop ER (−) mammary tumors spontaneously	N = 75 broods	Mothers treated with GE from 4 weeks of age until weaning of their calves (Ma-LT-GE), GE from gestation to weaning of their offspring (Ma-ST-GE), and offspring treated with GE postnatally (from 4 weeks of age to the end of the experiment) (Post-GE).	Ma-ST-GE: ↓ protection against breast cancer compared with Ma-LT-GE. Ma-LT-GE: ↑ expression of *Trp63*, ↓ than that of *Myc*. Results of chemoprevention similar to Post-GE.
Bowers et al., 2022 [20]	In vivo	C57BL/5 female mice	N = 100 broods	Mothers subjected to weight loss regimes 5 days a week for 10 weeks.	Caloric restriction: ↓ predominance to hypermethylation of DNA. Four of six genes that were differentially methylated and differentially expressed were also differentially expressed without being methylated.

BSps = broccoli sprouts; ER (−) = estrogen receptor negative; GE = genistein; HDACs = histone deacetylases; Ma-ST-GE = short-term maternal treatment with genistein; Ma-LT-GE = long-term maternal treatment with genistein; N = number; Post-GE = postnatal dietary exposure of offspring to genistein; SFN = sulforaphane; ↑ = increase; ↓ = decrease.

**Figure 3:**
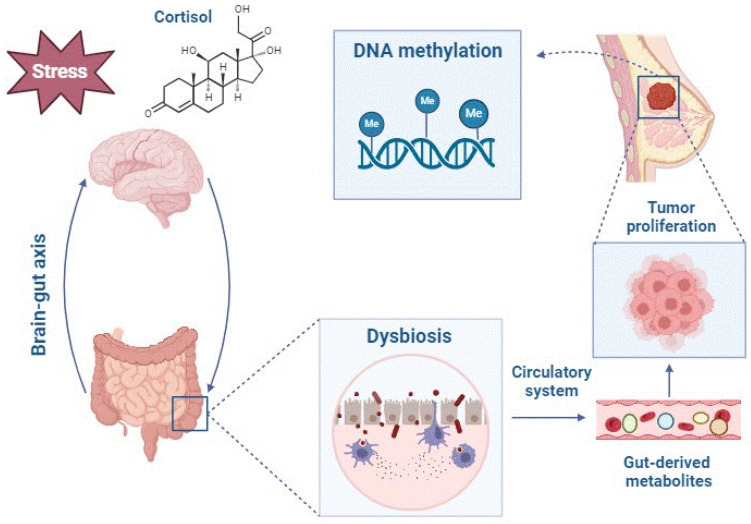
Altered gut-derived metabolites mediate epigenetic and gene expression changes in the breast tumor microenvironment, promoting breast cancer development.

Stress has been proposed as another active environmental factor modulating epigenetic regulation in breast cancer. Studies by Cui et al. (2022) [9] showed that stress induces gene expression and significant differential methylation of two genes, *Tbc1d9* and *Cdh10*, which are related to breast cancer prognosis and survival. The in vitro study carried out by Intabli et al. (2023) [3] to observe the influence of cortisol, a glucocorticoid released in stressful situations, on the development of breast cancer showed an epigenetic alteration characterized by decreased methylation levels in the promoter regions of several breast cancer suppressor genes (Figure 3 and Table 3).

The microbiota is considered an additional organ in the body. It has been observed that the composition of the gut and mammary microbiota may differ between patients diagnosed with breast cancer and healthy subjects due to a disruption in the balance of this commensal community (Table 4). This imbalance would result in an altered or dysbiotic state in the microbiota that could be implicated in the etiology of cancer, influencing the disease’s prevention, diagnosis, and prognosis [6,12,13,22]. 

**Figure 4:**
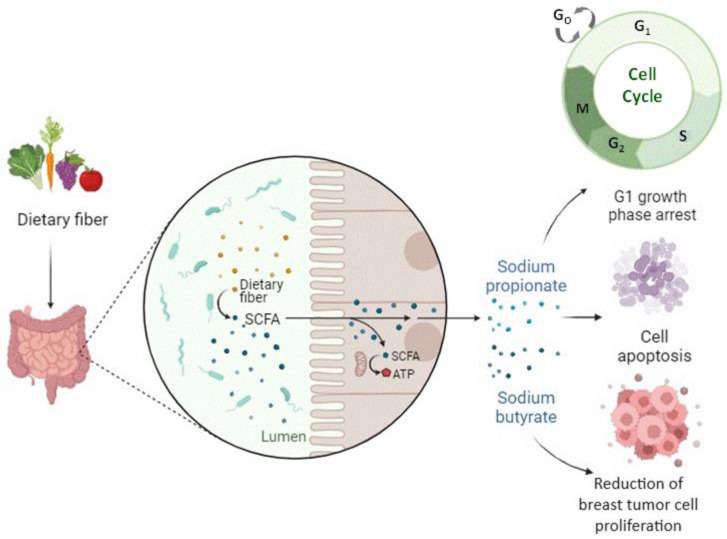
Short-chain fatty acids produced by the gut microbiota from the fermentation of dietary fibers positively impact the treatment and prevention of breast cancer.

It has recently been observed that one of the possible mechanisms by which the microbiota affects our health is interference with normal epigenetic control mechanisms. SCFAs produced by the gut microbiota from the fermentation of dietary fiber showed satisfactory results in the treatment and prevention of breast cancer, especially sodium butyrate (BS) and sodium propionate (PS), as reported by Semaan et al. (2020), Sharma et al. (2022), and Chen et al. (2022) [26] (Figure 4). Furthermore, according to Cui et al. (2022) [9], stress-induced taxonomic perturbations of the gut microbiome altered the metabolomic profile of intestinal and serum metabolites across the brain-gut axis, which are metabolites that may mediate epigenetic and gene expression changes at different locations in our body, including the mammary gland (Table 5).

## 4. Discussion

This systematic review synthesized evidence from 20 studies investigating the relationship between epigenetics, microbiota, and breast cancer. The analysis of the main results obtained indicates that dysbiosis and epigenetic modulations are risk factors in the development and aggressiveness of breast cancer.

A huge amount of relevant data regarding the role of epigenetic modulations in the induction of diseases, the role of the microbiome, and the exact mechanisms of interaction among them in disease pathogenesis have been reported in the last decade [28]. Concerning epigenetics, some authors have highlighted the link between epigenetic modifications and breast cancer. In 2024, Ou et al. reported that the methylation of GPRC5A promotes liver metastasis (the third most common occurrence in distant metastasis of breast cancer) and docetaxel resistance via the mTOR signaling pathway in TNBC [29]. In addition, the microbiota composition or microbiota-derived metabolites are also related to responses to immunotherapy, adverse events, and the heterogeneity of therapeutic effects. The mechanisms underlying gut microbiota-mediated potentiating efficacy while alleviating the side effects of immunotherapy generally differ across bacteria genera and immunotherapy types by enhancing anticancer immunity and modulating the tumor microenvironment [30]. Nevertheless, there is little research on the exact mechanisms by which the link between the microbiota and gene expression, and consequently the epigenetic pattern, affects the initiation and progression of breast tumors.

This review highlights the importance of and need for future research in this area, as it emphasizes the connection between the microbiota and the epigenetic pattern that influences the development of breast cancer, as well as the lack of scientific evidence and the limitations of existing scientific evidence. The main limitation of this work is the great variety between patient characteristics and experimental models, making it difficult to compare them.

### 4.1. Environmental Exposures, Such as Nutritional Status and Stress, Influence the Epigenetics of Breast Cancer

The results of studies focusing on the research of maternal diets based on BSps, GE, and canola oil, as well as calorie restriction, show the protective effect of these diets on offspring. As for maternal exposure to BSps, the well-documented suppressive effect of HDACs by the bioactive compounds abundant in this diet, such as SFN, is considered an important therapeutic pathway against cancer, since HDACs are involved in the repression of transcription through the removal of an acetyl group from the lysine residues of histone proteins. This can trigger chromatin condensation and lead to the silencing of genes responsible for regulating tumor suppression processes, leading to tumorigenesis. Similarly, changes in the expression of several tumor suppressor genes are negatively correlated with the enrichment of methylated histone H3K9, as it can impair chromatin accessibility and lead to inhibition of transcription. Increased DNA methylation throughout the genome may also indicate a mammary tumor suppressive effect, since global hypomethylation of genomic DNA contributes to genomic instability, abnormal chromosomal structure, and cellular transformation [19,21].

On the other hand, accumulating evidence shows the importance of the timing of soy consumption in its preventive effects on breast cancer [31]. Long-term maternal treatment with genistein (Ma-LT-GE) showed stronger and more prominent protective effects than short-term maternal treatment with genistein (Ma-ST-GE), confirming that maternal dietary exposure to GE determines the efficacy of breast cancer prevention in offspring. In addition, similar chemo-preventive results for Ma-LT-GE and postnatal dietary exposure of offspring to genistein (Post-GE) suggest that the protective effects of maternal diet GE can be transmitted to offspring and maintained in the next generations. Previous studies showed that the tumor-suppressive effect of GE was mediated by epigenetic mechanisms [32]. In this respect, genes such as *Myc* and *Trp63*, which may impact epigenetic mechanisms in breast cancer development, were identified, and gene expression and methylation changes were altered after Ma-LT-GE exposure [8]. We also observed a global demethylated tumor state in the offspring. However, although GE is thought to affect DNA methylation, the maternal GE diet did not affect DNMT activity or gene expression in the offspring, suggesting that maternal GE exposure may affect the epigenetic profile of offspring, which is attributed to its inhibitory effects on mammary tumors not through the regulation of DNMT expression or enzymatic activities but probably through epigenetic regulation inherited in early childhood [18].

Regarding maternal exposure to canola oil, some authors provided evidence of the effect of this maternal diet on genome-wide histone modifications in F1 offspring, which corroborates previous findings suggesting the involvement of transgenerational epigenetic processes in breast cancer prevention [33]. Consistent with these results, several studies have shown that diets rich in omega-3 fatty acids can inhibit the enzyme acetyl-CoA carboxylase, which would lead to an increase in the levels of CoA (the acetyl donor) and thus overall histone acetylation. The respective increase and decrease in H3K18ac Y H2K4me2 around the transcription start sites of overexpressed genes points to the driving role of epigenetic regulation on the beneficial effects of omega-3-rich maternal diets. These results are in line with recent studies that demonstrated the potential of omega-3 fatty acids as epigenetic modifiers [34]. On the other hand, it is likely that increased H3K18ac at transcription start sites activates important response genes to DNA damage and causes an early response against anthracene-induced tumorigenesis, which shows the possible role of this diet rich in omega-3 in the preparation of offspring to rapidly express DNA repair genes after exposure to a carcinogenic agent [7].

The results from Bowers et al. (2022) [20] demonstrated for the first time that weight loss through calorie-restricted regimens decreases obesity-promoted carcinogenic effects in TNBC mice. The observed obesity-associated DNA hypermethylation has been linked to pro-inflammatory pathways. Likewise, the significant decrease in hypermethylation in binding motifs for obesity-related transcription factors in a group subjected to the Mediterranean diet compared with the obese group is consistent with the findings of a previous study by the same authors which, using the same TNBC model, suggested that weight loss through bariatric surgery may have similar anti-inflammatory and methylation effects to those found in the Mediterranean diet [35]. On the other hand, the fact that four of the six genes differentially expressed were not differentially methylated indicates that the anti-tumor effects of the calorie restriction interventions cannot depend on their ability to reverse mammary epigenetic reprogramming.

Stress is another important factor addressed in this review, and it has been shown that cortisol released in stressful situations has been shown to alter the gene expression of key epigenetic markers in breast cancer, leading to modifications in the epigenome [3]. In addition, the increased *Cdh10* gene expression observed in stressful situations is associated with worse survival rates in breast cancer cases [9]. Regarding *Tbc1d9*, numerous studies suggest that its expression correlates inversely with tumorigenic potential and that its overexpression leads to a favorable prognosis [36].

### 4.2. The Microbiota Composition Could Be Used as a Potential Biomarker in the Diagnosis and Prognosis of Breast Cancer

The implications of the findings on microbial dysbiosis are highly relevant, as they could be used as a biomarker in the diagnosis and prognosis of breast cancer. The included literature analyzed the breast tissues of women diagnosed with breast cancer and showed results comparable to current evidence in terms of relative abundance and microbial diversity. The study by Hoskinson et al. (2022) [23] evaluated the role (not addressed in the literature thus far) of mammary microbiota in the earliest breast tumor development, discovering for the first time a unique signature of microbial composition that prevents the development of mammary breast neoplasms. Klan et al. (2020) [13] corroborated the key role of inflammation in the etiology of breast cancer.

In terms of gut microbial communities, it has been shown that, generally, as in the mammary microbiota, there is less uniformity in breast cancer patients. In addition, the data support the idea that inflammation, promoted by macrophage infiltration, precedes the development of aggressive breast cancer, suggesting that the accumulation of these macrophages is probably involved in enhancing dysbiosis-dependent dissemination of tumor cells to distal sites within the body [25]. Thus, with taxonomic differences in the microbiota of breast cancer patients, it is difficult to draw any conclusions due to the disparate results obtained in the different studies analyzed. The results also showed lower Firmicutes/Bacteroidetes (F/B) ratios in breast cancer patients. The F/B ratio is considered to play an important role in the maintenance of intestinal homeostasis, and an increase or decrease in this ratio is a hallmark of dysbiosis [6].

According to previous findings, *Clostridium* cluster XIVa and *Clostridium* cluster IV enrichment in patients with early-stage breast cancer were positively associated with tumor severity [37]. These gut bacteria have deconjugating enzymatic activity (β-glucuronidase and β-glucosidase) which allows them to catalyze the hydrolysis of glucuronidated estrogens and promote the reabsorption of their free active forms in the enterohepatic process, altering the systemic levels of estrogens. This event has been proposed as a possible connecting mechanism between the metabolic effects of the gut microbiota and the development of hormone-dependent breast cancer [12]. In contrast to these results, Caleça et al. (2023) [6] showed a significantly higher abundance of the genus *Clostridium* and *Escherichia coli*, both with β-glucuronidase activity, in healthy controls. These differences could be explained by the effect of treatment with chemotherapeutic agents (to which breast cancer patients were subjected in this study) on the gut microbiota, even though the contribution of chemotherapy to the smaller distribution of β-glucuronidase-producing bacteria remains to be clarified. On the other hand, the decrease in the relative abundance of *Coprococcus*, *Romboutisa*, *Butyricimonas*, *Odoribacter,* and *Akkermansia muciniphila* in women suffering from breast cancer could be related to the influence of these bacteria on the development of breast cancer since all five are producers of SCFA, and it is well known that SCFA exerts a modulatory role on cell proliferation, gene expression, apoptosis, and inflammation, which contributes to the enhancement of their antineoplastic effects [12,22].

The differences found in the microbial community structure of postmenopausal patients with breast cancer scheduled for adjuvant systemic treatment compared with neoadjuvant systemic treatment may be explained by the prophylactic use of antibiotics in adjuvant therapy. In the study conducted by Aarnoutse et al. (2021) [24], cefazolin, an antimicrobial active against a few Gram-negative bacteria, was used as a prophylactic antibiotic. It can be speculated that it does not directly affect *Dialister* or its corresponding family Veillonellaceae, which are Gram-negative stains. Thus, the enrichment of *Dialister* and Veillonellaceae, taxa associated with breast disease, in patients scheduled for adjuvant systemic treatment might be due to a cefazolin-induced reduction in other bacteria rather than an absolute increase. Also, the abundance of *Dialister* and Veillonellaceae was negatively correlated with the clinical stage and tumor size, being more advanced in patients scheduled for neoadjuvant therapy.

The maternal soy diet and stress exposure discussed above also modified the offspring’s gut microbiota GE-enriched maternal diet predominated in establishing the gut microbiota in early infancy. At the taxonomic level, the offspring of GE-treated dams showed a higher relative abundance of *Allobaculum*, considered an SCFA-producing microorganism, and *Bifidobacterium*, an important probiotic linked to intestinal homeostasis and gut health [36]. As a result, it has been suggested that offspring might “inherit” beneficial microbial species from the mother in utero or during lactation, which promotes their future health by maintaining a healthy and balanced bacterial ecosystem in the gut [18]. Stress increases the levels of Rhodospirillales and Clostridiales which, as indicated above, are related to the promotion of mammary carcinogenesis. Similarly, the F/B ratio decreased and, interestingly, saw a possible return of the abundance and composition of the microbiome to baseline or adaptation to a new state after stress, which could affect the pathways by which tumorigenesis is continuously promoted. In this sense, the composition of the gut microbiota exists in a dynamic equilibrium that would increase its complexity following exposure to stress [9].

The results of the investigated clinical trials showed a remarkable difference in the composition of the gut and breast microbiota of patients diagnosed with breast cancer compared with healthy subjects. One important limitation to consider regarding the clinical trials dealing with breast cancer and microbiota is that none of them addressed the question of whether the changes in the microbiota that occur in this disease are a cause or an effect of the disease. In this regard, our review only includes case-control studies, rather than cohort or nested case-control cohort studies, which means that the reported results may be due to reverse causality; that is to say, the development of breast cancer could cause the microbiota to change.

### 4.3. There Are Epigenetic Changes Sensitive to Alterations in the Microbiota That Are Associated with Breast Cancer

The role of SCFAs, particularly that of BS, has been studied over the years, especially in cases of colorectal cancer [38]. However, few studies have explored the effect of BS on breast cancer. Among them, Semaan et al. (2020) [26] investigated the role of BS and, for the first time, the role of PS in breast carcinogenesis and found that both SCFAs reduced the proliferation of MCF-7 cells, even though the effect of BS was greater compared with PS. This difference can be explained by the fact that the mechanism of histone hyperacetylation is more powerful in BS than in PS. BS has been described as a potent HDAC inhibitor. As a hypothesis, it has been suggested that the differences in its effect on HDACs may be explained by the fact that BS has a higher specificity for the active site of HDACs than PS. Concerning the cell cycle arrest triggered by low or medium levels of BS and PS, it could be explained by the decrease that these SCFAs could cause in the cell cycle regulatory proteins, which has been demonstrated concerning BS in previous studies conducted on colon carcinoma cells [39,40]. Furthermore, cell apoptosis induced after exposure to high levels of BS and PS has been described in other cancer cells, where BS and PS induced the activation of caspases 8 and 9, enzymes with a pro-apoptotic function in the extrinsic and intrinsic apoptosis pathways, respectively [40].

On the other hand, the tricombinant treatments of SFN, GE, and BS enhanced all these preventive effects against breast cancer. In addition, downregulation of the histone methyltransferases EZH2 and SUV39H1, observed after exposure to the combination of SFN, GE, and BS, is associated with reduced susceptibility to breast cancer [14]. This may be explained by the fact that EZH2 and SUV39H1 regulate gene expression through the transfer of methyl groups to amino acid residues of histones, and their positive regulation has been linked in previous studies to aggressive breast cancer and a poor prognosis for breast cancer survival [41].

The GE-based maternal diet and the combined BSp and green tea polyphenol (GTP) maternal diet also showed increased levels of beneficial metabolites produced by gut bacteria. In the case of maternal GE exposure, especially increased levels of butyrate, this SCFA acts as an epigenetic modulator that inhibits cell proliferation and negatively regulates tumor-promoting and key pro-inflammatory genes in breast cancer development [18]. In a maternal diet with BSps and GTPs, increased levels of isobutyrate, whose antineoplastic effects have been demonstrated in other studies on colon carcinoma [42] and propionate. These findings highlight the ability of maternal dietary interventions to alter epigenetic reprogramming during early embryogenesis and remodel the microbiota of the progeny through transplacental effects, leading to an improved health status and a more favorable breast cancer outcome in the adult life of the offspring [43].

Cui et al. (2022) [9] further hypothesized for the first time that stress may promote breast tumor initiation and progression through a stress-microbiome-metabolite-epigenetics-oncology axis. The observed results showed that stress-induced alterations in the gut microbiota may affect tumor growth at distal sites, in agreement with previous findings suggesting that the gut microbiome has endocrine functions [44]. The stress hormones, especially cortisol, altered the composition of the gut microbiome via the brain-gut axis, which substantially perturbed the metabolomic profile of intestinal and blood metabolites. These blood metabolites may subsequently mediate epigenetic and gene expression changes in the breast tumor microenvironment, promoting breast cancer development.

Finally, knowledge of the relationship between the microbiome and carcinogenesis would shed light on the mechanisms involved in the influence of microbial dysbiosis on cancer development and progression. These findings would contribute to advances in the potential use of the microbiome as a tool for prognostication and personalized therapy [45]. Furthermore, the reversible nature of epigenetic modulations, in contrast to genome mutations, and the ease of manipulation of our microbiome (e.g., by changing diet and stress, among other factors) open the door to the development of safe and effective therapeutic approaches for diseases as serious and widespread as breast cancer. Manipulation of both the microbiome and diet could lead to the development of new therapies that act through epigenetic pathways and add to our tools against cancer [28].

## 5. Conclusions

Twenty studies were analyzed, looking for connections between intestinal and mammary microbial communities, epigenetic modifications, and the development of breast cancer, and we found that both the microbiota and epigenetics play a key role in the onset and progression of breast cancer.

Environmental factors, as maternal dietary interventions with some bioactive compounds, exert transgenerational protective effects, providing an excellent chance to reverse dysregulated epigenetic profiles and inducing beneficial health outcomes to offspring. Stress is another environmental factor that affects breast cancer development through microbial and epigenetic modifications, and as a factor increasingly present in contemporary society, it is difficult to control.

The microbiota confers epigenetic protection against breast cancer through the production of SCFAs. Thus, PS and especially BS could be considered promising therapeutic agents for the treatment of breast cancer. On the other hand, microbial dysbiosis leads to taxonomic and relative abundance disturbances that could be used as a biomarker of breast cancer diagnosis, even though it is necessary to highlight the need for further studies due to the ambiguity of the taxonomic results obtained. Our results are also limited by the need for more qualified and precise scientific studies.

## Figures and Tables

**Table 1:** Population, Intervention, Comparison, and Outcome (PICO) criteria for inclusion of studies.

Parameter	Inclusion Criteria
Population	Studies performed in cells and animals, including humans (women) with breast cancer.
Intervention	Eligible interventions included tests associated with the Chao1 and Shannon indices in humans, the most widely used indices for quantifying species’ biodiversity and dietary interventions in animals.
Comparison	Healthy women and animals without breast cancer.
Outcome	Diagnostic accuracy of indices of microbial diversity and relative microbial abundance and the preventive effect of maternal dietary interventions.

**Table 3:** Summary of the studies addressing stress and epigenetic modifications in breast cancer.

Authors	Study Design	Model	Sample Size	Intervention	Key Findings
Cui et al., 2022 [9]	In vivo	BALB/c mice subjected to stress and not stressed	N = 12	Mice subjected to chronic stress by restriction 2 h per day for 10 consecutive days.	↑ expression of the *Cdh10* gene and ↓ expression of the *Tbc1d9* in TNBC, with ↓ survival and worse prognosis of breast cancer.
Intabli et al., 2023 [3]	In vitro	MDA-MB-231 (TNBC) y MCF-7 cells	N/R	Growth medium with fresh cortisol for 20 days.	↓ levels of methylation in the promoter regions of several tumor suppressor genes and loss of global DNA methylation.

N = number; N/R = not reported; TNBC = triple-negative breast cancer; ↑ = increase; ↓ = decrease.

**Table 4:** Summary of the studies on microbial dysbiosis and breast cancer..

Authors	Study Design	Sample	Sample Size	Method of Detection	Key Findings
Klann et al., 2020 [13]	Clinical trial Observational Case-control	Samples of bilateral breast tumors from patients with cancer and breast samples from healthy subjects.	N = 46	Sequencing of the hypervariable regions V1–V2 of the 16 s rRNA.	↑ uniformity and richness are bacterial in the normal breasts of healthy women.
Hoskinson et al., 2022 [23]	Clinical trial Observational Case-control	Samples of breast tumors from patients with cancer and breast samples from healthy subjects.	N = 159	Sequencing based on the 16 s rRNA gene.	↑ bacterial richness in samples of healthy breasts. The existence of a bacterial signature before the development of the tumor was maintained in cancerous tissues. ↓ functionality of the bacteriome in women with cancer.
Bobin-Dubigeon et al., 2021 [12]	Clinical trial Observational Case-control	Stool samples from female patients with breast cancer (early, untreated, before treatment) and stool samples from healthy subjects.	N = 55	Sequencing of the hypervariable regions V3–V4 of the 16 s rRNA.	↓ bacterial diversity, ↑ abundance of Firmicutes, *Clostridium* cluster XIVa, and *Clostridium* cluster IV, ↓ abundance of Bacteroidetes, *Butyricimonas* sp., *Odoribacter* sp., and *Coprococcus* sp. in breast cancer patients.
Caleça et al., 2023 [6]	Clinical trial Observational Case-control	Stool samples from female breast cancer survivors and stool samples from healthy controls.	N = 314	Sequencing of the amplicon of the V4 region of the 16 s rRNA gene.	Control group: ↑ bacterial diversity. ↑ F/B ratio, and abundance of *Clostridum perfringers*, *Escherichia coli*, and *Akkermansia muciniphila*.
Byrd et al., 2021 [22]	Clinical trial Observational Case-control	Stool samples from Ghanaian women diagnosed with breast cancer or non-malignant breast disease and stool samples from healthy women.	N = 895	Sequencing of the amplicon of the V4 region of the 16 s rRNA gene.	Different diversity in the control group. Bacteroidetes were consistently positively associated with breast cancer in contrast to *Romboutsia* and Coprococcus 2.
Aarnoutse et al., 2021 [24]	Clinical trial Observational Case-control	Stool samples from postmenopausal women with ER (+) breast cancer and stool samples from healthy women.	N = 148	Sequencing of the amplicon of the V4 region of the 16 s rRNA gene.	↑ abundance of Veillonellaceae and Dialister in patients scheduled for systematic adjuvant treatment.
Rosean et al., 2019 [25]	In vivo	C57BL/6 female mice.	N/R	Mice fed with an antibiotic cocktail for 14 days.	Dysbiosis eater preset: ↑ dissemination of tumor cells and ↑ inflammation in the breast tissue.
Chen et al., 2022 [18]	In vivo	SV40 female mice spontaneously developing mammary tumors and wild-type C57BL/6J female mice.	N = 30 broods	Mothers treated with GE from 4 weeks of age until weaning of their offspring.	Alteration of the gut microbial community and ↑ relative abundance of *Allobaculum*, *Bifidobacterium,* and Bacteroidetes in offspring.
Cui et al., 2022 [9]	In vivo	BALB/c mice subjected to stress and not stressed.	N = 12	Mice subjected to chronic stress by restriction 2 h per day for 10 consecutive days.	↓ F/B ratio and ↑ abundance of Rhodospirillales and Clostridiales.

ER (+) = estrogen receptor positive; F/B = Firmicutes/Bacteroidetes; N = number; N/R = not reported; ↑ = increase; ↓ = decrease.

**Table 5:** Summary of the studies addressing epigenetic modulations in microbiota-sensitive breast cancer.

Authors	Study Design	Model	Sample Size	Intervention	Key Findings
Semaan et al., 2020 [26]	In vitro	MCF-7 cell line	N/R	Incubation with BS or PS for 24–96 h at different concentrations.	BS effect was more powerful than PS. Low-to-medium levels of BS and PS: blockade of MCF-7 cells in G1. High levels of BS and PS: induced cell apoptosis in MCF-7.
Sharma et al., 2022 [14]	In vitro	Cell line MDA-MB-231 (TNBC) and MCF-7 (ER (+))	N/R	Individual and combined doses of GE, BS, and SFN for 3 days.	SFN, BS, and GE combined: ↓ enzymatic activity of HDACs and DNMTs, ↓ expression of EZH2 and SUVH39H1, and ↓ synergistically the cell viability in MCF-7 and MDA-MB-231.
Chen et al., 2022 [18]	In vivo	SV40 female mice that develop mammary tumors spontaneously and wild-type female C57BL/6J mice	N = 30 broods	Mothers treated with GE from 4 weeks of age until weaning of their offspring.	GE: ↑ SCFAs, ↓ tumor proliferation, and ↓ gene expression tumors.
Sharma et al., 2020 [27]	In vivo	Her 2/neu female mice	N = 120	Mice treated with BSps and GTPs from 3 weeks of age to adulthood and mothers treated with BSps and GTPs during their gestation and lactation.	Group nurtured from conception: ↑ isobutyrate and propionate.
Cui et al., 2022 [9]	In vivo	BALB/c mice	N = 12	Mice subjected to chronic stress by restriction 2 h per day for 10 consecutive days.	Stress caused taxonomic perturbations of the gut microbiome that altered the metabolomic profile of gut and serum metabolites.

BS = sodium butyrate; BSps = broccoli sprouts; DNMTs = DNA methylTransferases; ER (+) = positive estrogen receptor; Ge = genistein; GTPs = green tea polyphenols; HDACs = histone deacetylases; N = number; N/R = not reported; PS = sodium propionate; SCFAs = short-chain fatty acids; SFN = sulforaphane; ↑ = increase; ↓ = decrease.

## Data Availability

No new data were created.
